# Supplementary material for: COGEVIS: A New Scale to Evaluate Cognition in Patients with Visual Deficiency
Source: Behav Neurol. 2018 Jun 25;2018:4295184. doi: 10.1155/2018/4295184 (PMC6036847; doi:10.1155/2018/4295184)
Supplement: Supplementary Materials — The COGEVIS: a cognitive evaluation tool that does not rely on visual ability. The scale has a score range of 0–30 (higher scores indicating better function). [file 4295184.f1.docx]

COGEVIS

**Registration: / 3**

 "I will read you three words. Repeat them and try to hold them back because I will ask for them again"

1. Cigar / 1

2. Flower / 1

3. Door / 1

**Temporo-spatial orientation: / 5**

"I will ask you some questions about the date of the day and the place where we are."

1. What is the year? / 1

2. What is the month? / 1

3. What day of the week? / 1

4. What is the name of the hospital / clinic / facility where we are? / 1

5. What city are we in? / 1

**Attention and calculation: / 3**

"Count backwards from 100 by removing 7 each time until I stop you. 100-7 ... "

1. 93 / 1

2. 86 / 1

3. 79 / 1

**Language: / 8**

"I will ask you a few questions, try to answer as precisely as possible."

1. What object can give the time? (Accepted: watch, clock, pendulum) / 1

2. What object is used when it rains? (Accepted: umbrella, boots, raincoat) / 1

"Listen well and do what I am going to tell you"

3. Take the sheet of paper placed on the table in front of you with your right hand / 1

4. Fold it in half / 1

5. And throw it on the ground / 1

Lexical fluency: letter S (in 1 min):

"Name a maximum of different words starting with the letter S, for example animals, plants ... but no proper names (first names, names of cities or countries)".

Quotation details: 3: ≥ 10 words/ 2: 6 to 9 words/ 1: 3 to 5 words/ 0: 2 words or less / 3

**Praxis: / 3**

"How do you do with the hand to do ...".

1. Military Salute / 1

2. Send a kiss / 1

3. Drinking a glass / 1

**Touch Recognition: / 2**

"Now you're going to have to recognize objects that I'll put in your hand one by one. What is the name of this object? "

1. Coin / 1

2. Pen / 1

**Recall: / 3**

"Can you recall the words you had to remember earlier? ".

1. Cigar / 1

2. Flower / 1

3. Door / 1

**Executive functions: / 3**

Similarities: "how are they alike ..."

1. A banana and an orange? / 1

2. A table and a chair? / 1

3. A tulip, a rose and a daisy? / 1
